# Novel PCR markers for discriminating *RMia* resistance alleles in *Prunus* rootstock breeding

**DOI:** 10.3389/fpls.2026.1918119

**Published:** 2026-08-07

**Authors:** Beatriz Bielsa, Alba Ruiz, María J. Rubio-Cabetas, Jérôme Grimplet

**Affiliations:** 1 Departamento de Ciencia Vegetal, Centro de Investigación y Tecnología Agroalimentaria de Aragón, Avda, Zaragoza, Spain; 2 Instituto Agroalimentario de Aragón – IA2, CITA-Universidad de Zaragoza, Zaragoza, Spain

**Keywords:** deletion, genotyping, LTR retrotransposon insertion, marker-assisted selection (MAS), *Meloidogyne* spp., root-knot nematodes

## Abstract

Root-knot nematodes are pest of many plant species, particularly in southern Europe, where *Meloidogyne incognita*, *M. arenaria* and *M. javanica* are the most common species. In *Prunus* rootstock breeding, restrictions on nematicide use has increased importance of incorporating genetic resistance. Three resistance genes have been identified in *Prunus*: *Ma*, *RMja* and *RMia*. The latter is a TNL gene located on the linkage group 2 that confers resistance to *M. arenaria*, *M. incognita* and *M. ethiopica*. Resistance is expressed in both homozygous and heterozygous dominant genotypes, but not in homozygous recessive individuals. To improve the precision of existing markers and develop markers more closely associated with *RMia*, an *in silico* characterization of the genomic region containing this gene was conducted in six *Prunus* genotypes using ‘Texas’ almond reference genome v2.0 and a BAC sequence containing *RMia*. Comparative analysis identified, in the almond reference genome, the resistance-associated insertion previously reported in peach, characterized it as the LTR982 retrotransposon present only in the susceptible *RMia* haplotype. This previously described structural polymorphism was used to develop novel codominant molecular markers targeting conserved flanking regions. Among the primer pairs tested, three showed high discriminatory capacity. RTP-RMia-F3/R3 amplified a fragment in heterozygous and homozygous recessive individuals, but not in homozygous dominants. The ACI_9660_2 and NEM_6908_4 primers reliably distinguished resistant from susceptible genotypes. Together, these markers provide valuable tools for marker-assisted selection in *Prunus* rootstock breeding, enabling both resistance screening and allelic discrimination. The molecular results were further validated in hybrid rootstock genotypes through artificial inoculation assays.

## 1 Introduction

The Mediterranean basin is one of the main producing areas of stone fruit (*Prunus* spp.) thanks to its temperate climate, wide cultivars diversity, and increasing agricultural specialization. Spain stands as a major European producer, dedicating more than 700,000 ha to the cultivation of these crops (FAO, 2025). The Guadalquivir Valley accounts for 31% of this area and represents the largest almond-growing region in the country (MAPA, 2025). However, this region faces significant constraints, including problems associated with the presence of soil pathogens such as root-knot nematodes (RKN; *Meloidogyne* spp.), which severely threaten plantation productivity (Clavero-Camacho et al., 2024).

RKN are among the most destructive soil-borne pests in *Prunus* spp. distributed worldwide (Jones et al., 2013), especially in sandy soils, inducing root galls, defoliation, compromising water and nutrient uptake, and ultimately reducing tree vigor and productivity, sometimes leading to the tree death (Saucet et al., 2016; Esmenjaud, 2021; Clavero-Camacho et al., 2024). Main RKN species affecting and damaging *Prunus* spp. in the Mediterranean Basin include *M. arenaria*, *M. javanica* and *M. incognita* (Esmenjaud, 2021). Other species have been identified in different areas and crops, among them, *M. enterolobii*, which is a highly aggressive species (Kiewnick et al., 2008), *M. ethiopica* affects mainly in South America (Meza et al., 2016), *M. floridiensis* in USA (Handoo et al., 2026), *M. morocciencis* in Morocco (Rammah and Hirschmann, 1990) and *M. hispanica* in southern Spain (Hirschmann, 1986).

Historically, nematode-related damage in agriculture was primarily managed through chemical control methods. However, the use of nematicides has progressively forbidden, giving way to more economically and environmentally sustainable alternatives based on an integrated nematode management strategy that combines biological and cultural approaches, with particular emphasis on the selection of resistant germplasm (Sikora et al., 2023). Consequently, the identification and development of nematode-resistant rootstocks becomes a critically important issue to ensure plantation health and contribute to the sustainability and competitiveness of the sector in a highly demanding environment.

In previous reports, the variability of the RKN resistance has been demonstrated, allowing its incorporation in new materials (Pinochet, 1997). Recent studies have explored the genetic bases of resistance, finding natural sources of RKN resistance exist in *Prunus* germplasm and three major dominant resistance (R) genes have been identified and mapped in *Prunus* species: *Ma*, *RMja* and *RMia* genes, that represent the main sources of resistance characterized to date (Rubio-Cabetas et al., 2000; Claverie et al., 2004, 2011; Duval et al., 2013, 2019; Van Ghelder et al., 2018). *Ma* from Myrobalan plum (*P. cerasifera*) was associated with a wide spectrum of resistance against *M. arenaria*, *M. incognita* and *M. javanica*, as well as others, including *M. ethiopica*, *M. floridensis* and *M. enterolobii*. This gene, located in chromosome 7 of the peach reference genome, belongs to the TNL (toll interleukin 1 receptor nucleotide-binding leucine-rich repeat) class of resistance genes, one of the most widely distributed gene families in plants (Claverie et al., 2011; Duval et al., 2019). The *RMja* gene was first identified in the ‘Alnem’ rootstock series, was described later than *Ma*, and is also located in chromosome 7. It is, in fact, likely the almond ortholog of the *Ma* gene. This gene confers resistance to *M. arenaria*, *M. javanica*, *M. ethiopica*, and *M. enterolobii* (Van Ghelder et al., 2018). The third gene, *RMia*, is a putative TNL (TIR-NBS-LRR) gene described in ‘Nemaguard’ and ‘Nemared’ peaches and located in chromosome 2 of the peach reference genome (Claverie et al., 2004; Duval et al., 2013). *RMia* gene controls the resistance of *M. arenaria*, *M. incognita* and *M. ethiopica* (Duval et al., 2019). Genetic studies demonstrated that resistance is conferred by the dominant homozygous and heterozygous genotypes, but not by the recessive homozygous genotype (Claverie et al., 2004).

Although the identification of *RMia* gene constituted a significant advance in *Prunus* breeding, the efficient implementation of this resistance within marker-assisted selection (MAS) programs has been constrained by the availability and accuracy of linked molecular markers. In this context, a high-resolution genetic analysis conducted in ‘Nemared’ enable a more precise delimitation of *RMia* region (Duval et al., 2013). This approach identified several flanking SSR and SNP markers and confined the locus to an approximately 92 kb genomic sequence harboring four candidate TIR-NBS-LRR genes. Within that interval, three markers (SNP_APP92, SNP_APP91, and AMPP117) displayed a strong genetic association with the resistance phenotype, co-segregating with the resistance in most of the *Prunus* accessions analyzed (Duval et al., 2013). Nevertheless, since these markers are positioned in regions flanking the gene rather than within the functional sequence itself, the occasional recombination events between marker loci and *RMia* gene may disrupt their association. This limitation underscores the importance of developing gene-targeted markers that are physically anchored within the *RMia* locus to ensure more reliable and accurate selection results. More recently, Duval et al. (2025) characterized the resistant *RMia* haplotype in peach by identifying three candidate TNL genes (*RMiaGC1*, *RMiaGC2* and *RMiaGC3*) involved in the resistance to *M. incognita*, with *RMiaGC1* proposed as the most likely candidate gene. Their analysis revealed a large insertion within the central region of *RMiaGC1* in the susceptible haplotype, whereas this insertion was absent from the resistant haplotype. Based on these findings, they developed intragenic markers within *RMiaGC1*, G13F1R1 and G13QP211, and RMiaGC2, G31F5R5, and confirmed SNP_APP92 as a robust marker associated with *RMia* resistance. The combined use of these markers showed a good concordance between marker genotypes and resistance phenotypes, confirming their suitability for MAS in peach breeding programs (Duval et al., 2025).

Building upon the evidence that this structural variant distinguishes the resistant and the susceptible *RMia* haplotypes, we further investigated the organization of this genomic region using an alternative genomic framework based on the ‘Texas’ almond reference genome (Alioto et al., 2019). By comparing both haplotypes within the almond genomic background, we aimed to refine the structural characterization of the insertion and develop novel codominant markers directly targeting this polymorphism. This strategy was intended to improve marker specificity and facilitate the development of diagnostic tools capable of more precise allelic discrimination at the *RMia* locus in segregating interspecific hybrid populations derived from *Prunus* rootstock breeding programs.

## 2 Materials and methods

### 2.1 Plant materials

In this study, four sets of genotypes were considered according to the stage of the analysis (**Table 1**): (i) *in silico* analysis of the genomic region containing the *RMia* gene; (ii) reference genotypes used for the preliminary validation of primers designed within the *RMia* gene region; (iii) advanced breeding selections and their parental lines to assess marker applicability within the breeding program; and finally, (iv) genotypes previously phenotyped for resistance to *Meloidogyne incognita*, *M. arenaria*, and *M. javanica* for the functional validation of the developed markers.

**Table 1:** Plant material used in different stages of marker development and validation.

Accessions	*Prunus* species	Phenotype (*Mi - Mj - Ma*)	*RMia* genotype
*In silico* sequence analysis			
Lauranne	*P. dulcis*	S - S - S	*rmia/rmia*
Alnem	*P. dulcis*	S - S - S	*rmia/rmia*
Garfi	*P. dulcis*	S - S - S	*rmia/rmia*
Nemared	*P. persica*	R - R/S - R	*RMia/RMia*
Garnem	*P. dulcis × P. persica*	R - S - R	*RMia/rmia*
ACI-150803-20	*P. persica × P. kuramica*	R - S - R	*RMia/RMia*
Preliminary validation			
Garfi	*P. dulcis*	S - S - S	*rmia/rmia*
Nemared	*P. persica*	R - R/S - R	*RMia/RMia*
Garnem	*P. dulcis × P. persica*	R - R/S - R	*RMia/rmia*
Felinem	*P. dulcis × P. persica*	R - R/S - R	*RMia/rmia*
Pilowred^®^	*P. dulcis × P. persica*	R - S - R	*RMia/rmia*
Myrobalan P. 2175	*P. cerasifera*	R - R - R	*Ma*/ma^*^
Advanced breeding genotypes and parental lines			
Parental 08	*P. persica*	R - R/S - R	*RMia/RMia*
Parental 03	*P. kuramica*	ND	ND
ACI-150803-11	*P. persica × P. kuramica*	ND	ND
ACI-150803-13	*P. persica × P. kuramica*	ND	ND
ACI-150803-20	*P. persica × P. kuramica*	R - S - R	*RMia/RMia*
ACI-170803-74	*P. persica × P. kuramica*	ND	ND
ACI-170803-75	*P. persica × P. kuramica*	ND	ND
ACI-190803–02 to ACI-190803-17^¤^	Progeny 08 × 03	ND	ND
Parental 14	*P. dulcis* × (*P. persica* × *P. davidiana*)	ND	*RMia/rmia*
Parental 33	*P. dulcis × P. persica*	R - R/S - R	*RMia/rmia*
ACI-181433–02 to ACI-201433-30^‡^	Progeny 14 × 33	ND	ND
Parental 32	*P. persica*	R - R/S - R	*RMia/RMia*
Parental 02	*P bucharica*	ND	ND
ACI-183202–01 to ACI-183202-04^§^	Progeny 32 × 02	ND	ND
Functional validation			
ACI-150803-11	*P. persica × P. kuramica*	R - R/S - R	ND
ACI-150803-20	*P. persica × P. kuramica*	R - S - R	*RMia/RMia*
ACI-150803-04	*P. persica × P. kuramica*	R - R - R	ND
ACI-151406-07	*P. dulcis* × (*P. persica* × *P. davidiana*) × *P. feznliana*	R - R - R	ND
ACI-163230-03	*P. persica × P. webbii*	R - S - R	ND
ACI-190803-04	*P. persica × P. kuramica*	R - R - R	ND
ACI-171302-13	[(*P. davidiana* × *P. persica*) × *P. persica*] × *P. bucharica*	R - S - R	ND
ACI-184742-02	(*P. cerasifera* × *P. bucharica*) × *P. dulcis*	R - R - R	ND

Resistance phenotypes against *Meloidogyne incognita* (*Mi*), *M. javanica* (*Mj*), and *M. arenaria* (*Ma*) are indicated when available. *RMia* genotypes correspond to previously characterized genotypes or genotypes inferred from marker analyses, ND: *RMia* genotype not determined prior to molecular characterization in this study. .

^*^Genotype carrying the *Ma* gene.

^¤^Advanced selections derived from the cross Parental 08 × Parental 03.

^‡^Advanced selections derived from the cross Parental 14 × Parental 33.

^§^Advanced selections derived from the cross Parental 32 × Parental 02.

### 2.2 *In silico* sequence analysis

The genomic region containing the *RMia* gene was analyzed using six *Prunus* genotypes representing the three genotypic classes: the homozygous resistant genotypes ‘Nemared’ and ‘ACI-150803-20’ (*RMia*/*RMia*); the heterozygous resistant rootstock ‘Garnem’ (*RMia*/*rmia*); and homozygous susceptible almond cultivars ‘Lauranne’, ‘Alnem’ and ‘Garfi’ (*rmia*/*rmia*) (**Table 1**). Whole-genome sequencing data from these genotypes, generated in earlier studies, were aligned against a custom reference genome composed of the almond reference genome ‘Texas’ v2.0 (Alioto et al., 2019) together with a BAC sequence containing the *RMia* locus (Duval et al., 2025). Sequence alignments were performed using tools available on the Galaxy Europe platform and visualized using the Integrative Genomics Viewer (IGV) software (Robinson et al., 2011).

Additionally, whole-genome sequencing reads previously generated in earlier studies for the resistant genotypes ‘ACI-150803-20’ and ‘Nemared’ were *de novo* assembled using SPAdes (Prjibelski et al., 2020) to characterize the genomic structure of the resistant genotypes and facilitate the design of resistance-associated molecular markers.

### 2.3 Transposon insertion identification and primer design

Based on the identified transposon insertion polymorphism identified within the *RMia* candidate region, several primer pairs were designed to discriminate between resistant and susceptible genotypes. Primer design focused on regions located upstream, downstream and overlapping the insertion site in order to maximize amplification specificity and allelic discrimination among the homozygous resistant, and homozygous susceptible genotypes.

Special attention was given to the retrotransposon-associated insertion identified within the NBS domain of susceptible genotypes, as this polymorphism represented a potential functional marker directly associated with the resistance locus.

All primer sets were designed using Primer3web v.4.1.0 (Untergasser et al., 2012), with default parameters, and the resulting primer combinations were subsequently evaluated by PCR amplification in the different genotype sets described in **Table 1**. The selected primers sequences and expected amplicon sizes are detailed in **Table 2**.

**Table 2:** Characteristics of the three molecular markers developed for the *RMia* locus.

Primer name	5’ → 3’ sequence	Tm annealing	PCR product size (bp)
RTP-RMia_F3	GGCGCCAATGCATTACTTAT	59.96	188
RTP-RMia_R3	TCCGAAGCACAAAGAACACA	60.43	
ACI_9660_2_Fwd	ACCATAATGCCCTACAGCTATC	57.44	208
ACI_9660_2_Rev	CGAGAAGGGAGCGTATGTTTCT	60.16	
NEM_6908_4_Fwd	GGAAATGATCAAAATCGCACCCT	59.87	568
NEM_6908_4_Rev	TTGGACATATTAGGAAGTGGCGT	59.8	

### 2.4 DNA isolation and PCR amplification

Genomic DNA was extracted from approximately 0.05 g of young leaf tissue from the *Prunus* genotypes listed in **Table 1** using the CTAB protocol described by (Doyle and Doyle, 1987). DNA quality and concentration were assessed using a Nanodrop^®^ UV-Vis spectrophotometer (Nanodrop Technologies, Wilmington, DE, USA).

PCR amplifications were performed in a final reaction of 20 µL containing 40 ng of genomic DNA, 0.25 µM of each primer, and Taq DNA polymerase according to the manufacturer’s recommendations (Invitrogen, ThermoFisher Scientific). Amplifications were carried out using the following thermal profile: an initial denaturation step at 95°C for 4 min; followed by 37 cycles of 95°C for 30 s, 60°C for 45 s, and 72°C for 1 min, and a final extension step at 72°C for 10 min.

PCR products were separated by electrophoresis on 1.2% (w/v) agarose gels, stained with GelRed^®^, and visualized under UV illumination.

### 2.5 Phenotypic evaluation of the RKN resistance

To validate the molecular results obtained with the developed markers, eight genotypes from the CITA *Prunus* breeding program were phenotypically evaluated for resistance to root-knot nematodes (**Table 1**). The commercial rootstocks ‘GF-677’, ‘Garnem’ and ‘Adara’ were included as reference controls. Phenotypic evaluations were performed only in advanced breeding genotypes previously identified as putatively resistant, as the objective of the assay was to confirm resistance by phenotyping before those advancing selections go to the next stage of the breeding evaluation. Susceptible genotypes were not included because they had already been discarded during the breeding selection and high inoculation pressure method assays are labor-intensive and costly.

Artificial inoculations were conducted at the Laboratorio de Sanidad Vegetal de la DTEVL del CSIC-INIA as an external specialized service following a protocol adapted from the CPVO-TP 044/04 guidelines originally established for tomato (CPVO, 2021). The *Meloidogyne* species used for the assays were routinely identified and maintained by the laboratory. Independent inoculation assays were performed for each of the three nematode species analyzed: *Meloidogyne arenaria*, *M. incognita* and *M. javanica*.

For each genotype and nematode species, ten one-year-old plants (five biological replicates consisting of two plants each) were evaluated. To ensure a high inoculum pressure throughout the experiment, each replicate growth with one susceptible tomato plant at the four-leaf stage inoculated previously with 2 g of root tissue heavily infected with the corresponding *Meloidogyne* species. Non-inoculated control plants were also included for each genotype. After 40 days, the tomato plants were replaced with newly inoculated susceptible tomato plants to maintain high inoculum pressure during the 180-day evaluation period.

After 180 days from the first inoculation, root systems were washed and examined for gall formation. Root galling was assessed using a 0–4 galling index adapted from standard *Meloidogyne* evaluation scales (Barker, 1985), where 0 = absence of galls; 1 = less than 25% of roots with galls; 2 = 25-50% of roots with galls; 3 = more than 50% of roots with galls; and 4 = severe root galling generally associated with plant death. Based on these scores, plants were classified as resistant (score 0), moderately resistant (scores 1-2), or susceptible (scores 3-4). Phenotypic responses were subsequently compared with the genotypic profiles obtained using the developed molecular markers to assess marker-trait association.

Because the objective of these assays was to validate the correspondence between molecular marker genotypes and resistance phenotypes, phenotypic responses were evaluated descriptively according to the resistance classes defined above. Marker performance was assessed qualitatively based on the agreement between phenotypic classifications and marker-inferred genotypes. No inferential statistical analyses were performed.

## 3 Results and discussion

The identification of molecular markers tightly associated with resistance loci remains a major objective in *Prunus* rootstock breeding programs because they facilitate early selection and reduce the need for labor-intensive phenotypic screening. In the case of root-knot nematode (RKN) resistance, several markers linked to the *Ma*, *RMja* and *RMia* loci have been developed during the last two decades (Claverie et al., 2004; Van Ghelder et al., 2018; Duval et al., 2019, 2025). However, the exploration of structural variation within candidate resistance regions remains largely unexplored.

### 3.1 Identification of structural polymorphisms within the RMia locus

Comparative analysis of the aligned genomic sequences revealed the presence of a large transposon insertion polymorphism within the genomic interval flanked by the SNP markers SNP_APP92 and KASP_A26, previously reported as closely linked to the *RMia* gene region (Duval et al., 2016). Structural annotation of this region identified the insertion as an LTR982 retrotransposon, located within the candidate *RMia* interval and mapping to the genomic region corresponding to the TIR-NBS-LRR gene.

TIR-NBS-LRR genes belong to the major class of plant resistance (*R*) genes and represent an important source of genetic variation for the development of resistance-associated molecular markers (Marone et al., 2013). In *Prunus*, several RKN resistance genes belong to this family, including the broad-spectrum resistance gene *Ma* from *P. cerasifera* (Claverie et al., 2011) and the *PKMi* locus identified in *P. kansuensis* (Cao et al., 2011).

**Figure 1:**
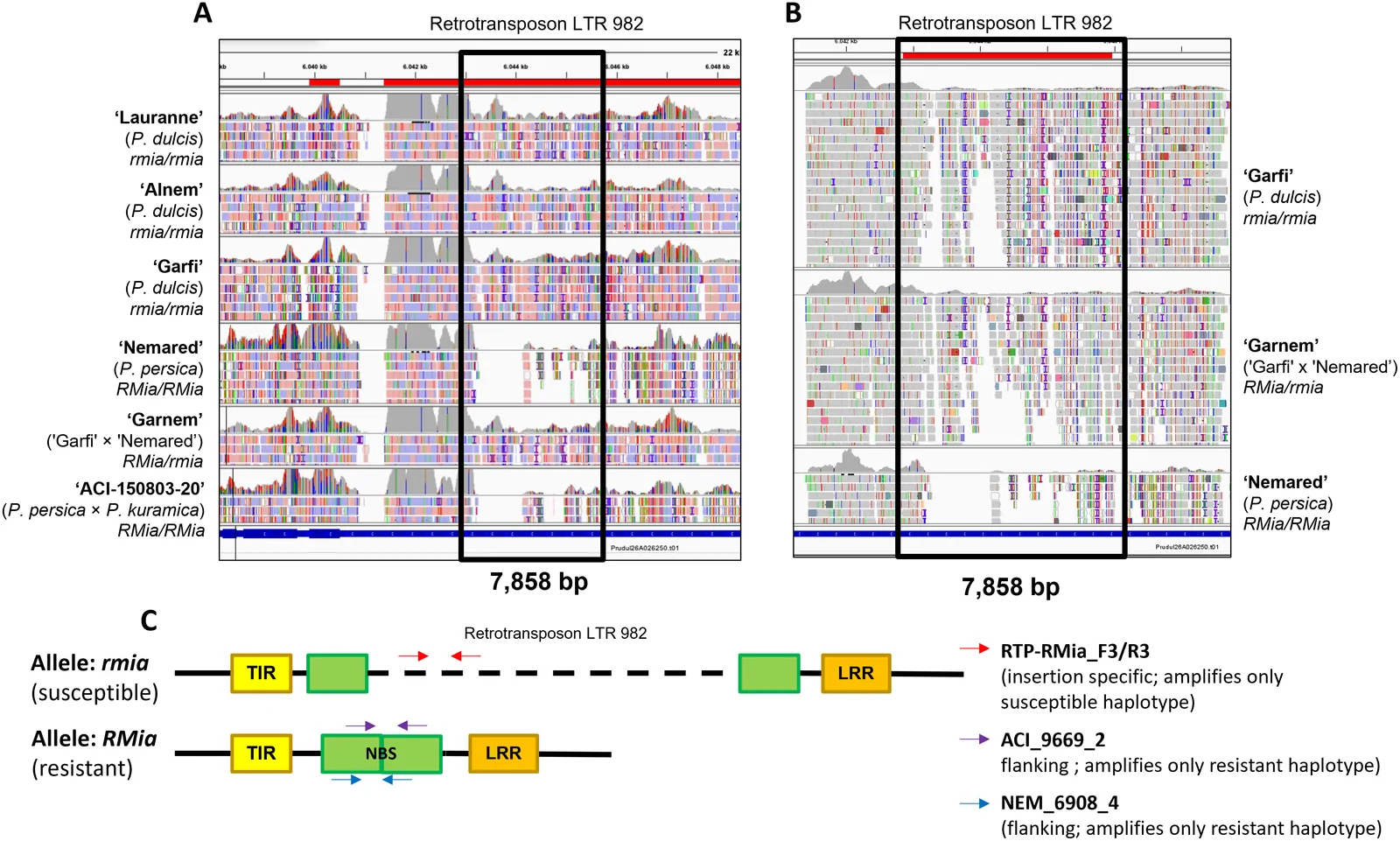
Identification of an LTR retrotransposon insertion and design of allele-specific markers for the *RMia* locus. **(A)** IGV visualization of sequence alignments from resistant and susceptible *Prunus* genotypes across the *RMia* candidate region. The 7,858 bp insertion corresponding to the LTR retrotransposon 982 (LTR982) is present in susceptible genotypes (*rmia*/*rmia*) and absent in resistant genotypes (*RMia*/*RMia*). The heterozygous rootstock ‘Garnem’ (*RMia*/*rmia*) shows an intermediate sequence coverage across the insertion region. **(B)** Detailed view of the LRT982 in ‘Garfi’, ‘Garnem’ and ‘Nemared’, illustrating the reduced coverage observed in the heterozygous genotype. **(C)** Schematic representation of the resistant (*RMia*) and susceptible (*rmia*) haplotypes showing the position of the LTR982 insertion within the candidate *TNL* gene and the relative locations of the three primer pairs. The susceptible allele contains the 7,858 bp insertion within the NBS domain, whereas the resistant allele lacks the insertion and remains an intact NBS-LRR structure.

The retrotransposon insertion was consistently associated with the susceptible *RMia* haplotype and was absent from the resistant haplotype. Specifically, the insertion was identified in the almond genotypes ‘Lauranne’, ‘Alnem’ and ‘Garfi’, all previously characterized as homozygous susceptible (*rmia*/*rmia*), whereas it was absent for the resistant genotypes ‘Nemared’ peach and the hybrid selection ‘ACI-150803-20’ (*RMia*/*RMia*) (**Figure 1A**). In heterozygous rootstock ‘Garnem’ (*RMia*/*rmia*), derived from the cross ‘Garfi’ × ‘Nemared’, also displayed the retrotransposon-associated region, although sequence coverage across the insertion was approximately half that observed in homozygous susceptible genotypes (**Figure 1B**), indicating that the retrotransposon is present in only one of the two haplotypes, as expected for a heterozygous genotype.

The insertion spans approximately 8kb, between positions 6,043,182 bp and 6,051,040 bp of the ‘Texas’ almond reference genome v2.0. This structural polymorphism corresponds to the resistant-associated insertion previously reported by Duval et al. (2025) within the *RMia* candidate region. However, the use of the almond reference genome together with the *RMia* BAC sequence enabled its structural characterization as an LTR982 retrotransposon and allowed the comparison of the resistant and susceptible haplotypes. Unlike previously reported markers, which were developed from polymorphisms identified within the resistant peach haplotype, the present study exploits a structural variant distinguishing both haplotypes in the almond genomic background to develop a novel codominant marker system.

Based on this structural polymorphism, the genomic regions flanking the retrotransposon insertion were compared among the resistant genotypes (‘ACI-150803-20’ and ‘Nemared’) and the susceptible ‘Texas’ almond reference genome. Two conserved regions of approximately 560 bp, located upstream and downstream of the insertion site, respectively, were identified across the analyzed genomes. These conserved flanking regions were subsequently used for primer design to discriminate resistant and susceptible genotypes and to differentiate homozygous and heterozygous individuals.

### 3.2 Development and preliminary validation of RMia-derived molecular markers

Based on the structural polymorphism identified within the *RMia* candidate region, several primer combinations were designed to amplify genomic regions flanking or overlapping the LTR982 insertion. Primer design focused on generating diagnostic amplification profiles to distinguish resistant and susceptible genotypes while also enabling the identification of homozygous and heterozygous individuals.

**Figure 2:**
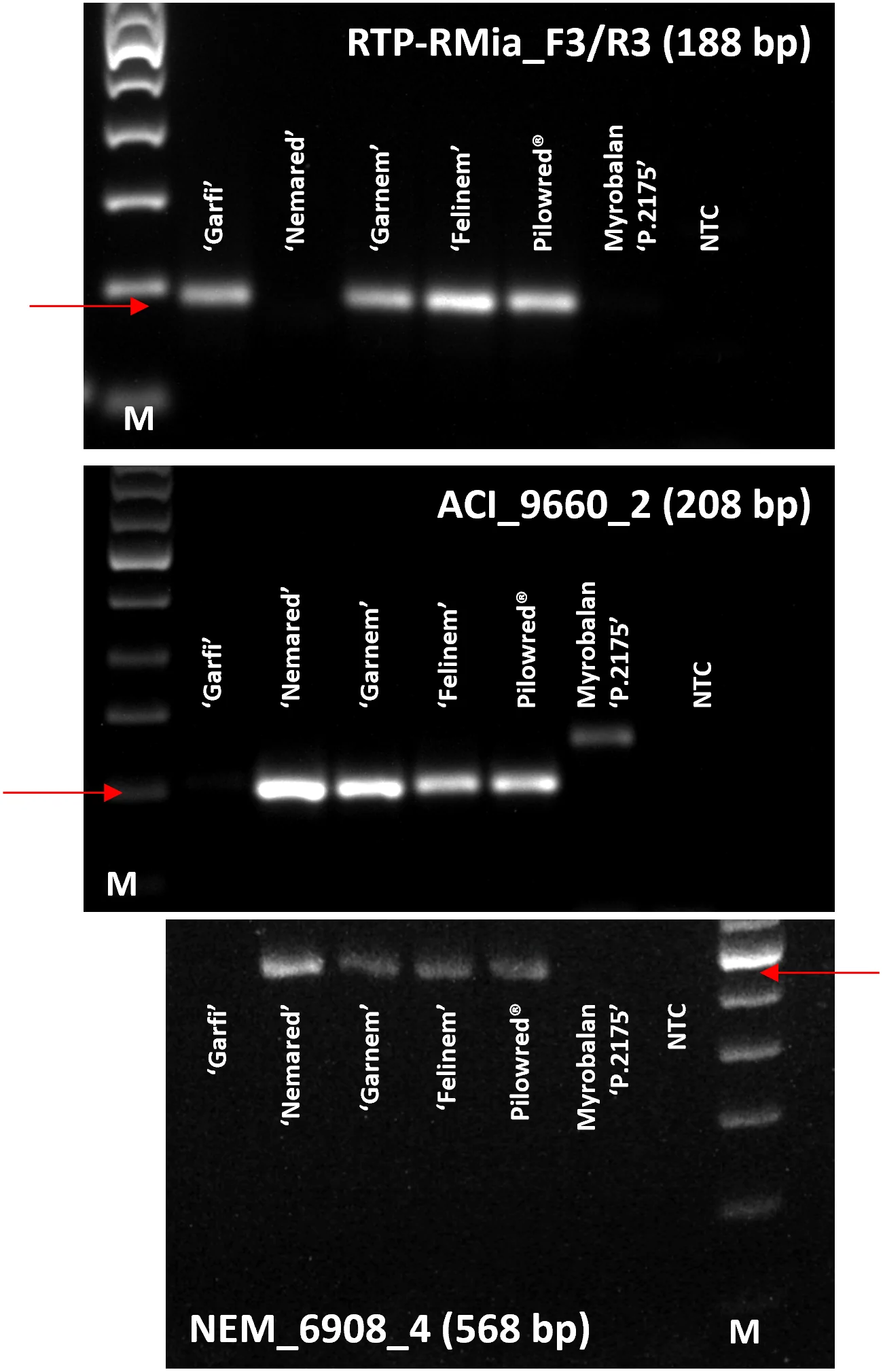
PCR amplification patterns generated by the *RMia* markers during preliminary validation in reference *Prunus* genotypes with known *RMia* genotypes. The three amplification profiles correspond to independent agarose gels obtained with the primer combinations RTP-RMia_F3/R3, ACI_9660_2, and NEM_6908_4. M: 100 bp DNA ladder. NTC: non-template control. The red arrows indicate the expected PCR products (188, 208 and 568 bp, respectively).

A total of 14 primer pairs were initially evaluated using representative resistant and susceptible genotypes. Among them, the primer combinations RTP-RMia_F3/R3, ACI_9660_2 and NEM_6908_4 (**Table 2**) generated the most consistent and reproducible amplification patterns and were therefore selected for subsequent validation analyses. Amplification profiles differed according to the allelic composition of the *RMia* locus, with resistant genotypes showing the expected amplicons corresponding to the haplotype lacking the LTR982 insertion for ACI_9660_2 and NEM_6908_4, whereas genotypes carrying the susceptible *RMia* haplotype (*rmia* allele), including heterozygous individuals, amplified the insertion-containing region using RTP-RMia_F3/R3 primer combination (**Figure 2**). The Myrobalan ‘P.2175’ plum was included as a negative control because it carries the *Ma* resistance locus instead of *RMia*.

In heterozygous genotypes such as ‘Garnem’, amplification profiles corresponding to both resistant and susceptible alleles were detected, confirming the co-segregation of the two allelic variants and supporting the codominant behavior of the developed markers (**Figure 2**). The ability to discriminate homozygous resistant, heterozygous resistant and homozygous susceptible genotypes highlights the usefulness of these markers for breeding applications.

The allele specificity of the markers is determined by their position relative to the LTR982 insertion. RTP-RMia_F3/R3 amplifies the susceptible haplotype because the primers anneal within the retrotransposon, whereas ACI_9660_2 and NEM_6908_4 span the insertion site and therefore generate amplification only in the absence of the insertion corresponding to the resistant haplotype.

Previous studies on the *RMia* locus have mainly focused on the identification of linked SNP markers (Duval et al., 2013). More recently, Duval et al. (2025) characterized resistance and susceptible *RMia* haplotypes and developed several intragenic markers within the candidate genes *RMiaGC1* and *RMiaGC2*.These markers showed complete correspondence with the resistant phenotype in a diverse panel of peach and peach-derived rootstocks. However, they were dominant in nature and did not allow discrimination between homozygous resistant and heterozygous resistant individuals, as both genotypic classes produced identical amplification profiles.

Unlike previously reported markers, which were developed from polymorphisms identified in the peach *RMia* candidate region, the markers described here exploit a structural polymorphism, distinguishing the resistant and susceptible haplotypes identified in ‘Texas’ almond reference genome. As a result, they not only discriminate the resistant and susceptible genotypes but also distinguish resistant and heterozygous resistant individuals, providing a fully codominant marker system for the *RMia* locus.

The ability to determine allele zygosity at the *RMia* locus is particularly valuable in almond × peach rootstock breeding programs, where heterozygous resistant individuals are frequently used as parents. Because the markers target a structural polymorphism located within the candidate resistance interval, they are also expected to be less affected by recombination than markers located outside the locus (Salgotra and Neal Stewart, 2020). Together, these characteristics make them useful tools for marker-assisted selection (MAS) and resistance gene pyramiding.

### 3.3 Validation in breeding germplasm and parental lines

The applicability of the markers developed for MAS was evaluated in advanced breeding germplasm and their corresponding parental lines (**Table 3**). The three markers identified the expected allelic combination of the parental genotypes and enabled the classification of the progenies into homozygous resistant, heterozygous resistant and homozygous susceptible classes.

**Table 3:** Genotyping results obtained with the three *RMia* markers in advanced breeding selections and their parental lines.

Genotype	RTP-RMia_F3/R3	ACI_9660_2	NEM_6908_4	*RMia* genotype
Progeny 08 × 03				
Parental 08	-	+	+	*RMia/RMia*
Parental 03	-	-	-	ND
ACI-150803-11	-	+	+	*RMia/RMia*
ACI-150803-13	+	+	+	*RMia/rmia*
ACI-150803-20	-	+	+	*RMia/RMia*
ACI-170803-74	+	+	+	*RMia/rmia*
ACI-170803-75	+	+	+	*RMia/rmia*
ACI-190803-02	-	+	+	*RMia/RMia*
ACI-190803-03	-	+	+	*RMia/RMia*
ACI-190803-04	-	+	+	*RMia/RMia*
ACI-190803-05	-	+	+	*RMia/RMia*
ACI-190803-06	+	+	+	*RMia/rmia*
ACI-190803-07	+	+	+	*RMia/rmia*
ACI-190803-08	-	+	+	*RMia/RMia*
ACI-190803-09	-	+	+	*RMia/RMia*
ACI-190803-10	-	+	+	*RMia/RMia*
ACI-190803-11	-	+	+	*RMia/RMia*
ACI-190803-12	+	+	+	*RMia/rmia*
ACI-190803-13	+	+	+	*RMia/rmia*
ACI-190803-14	-	+	+	*RMia/RMia*
ACI-190803-15	-	+	+	*RMia/RMia*
ACI-190803-16	-	+	+	*RMia/RMia*
ACI-190803-17	-	+	+	*RMia/RMia*
Progeny 14 × 33				
Parental 14	+	+	+	*RMia/rmia*
Parental 33	+	+	+	*RMia/rmia*
ACI-181433-02	+	-	-	*rmia/rmia*
ACI-181433-05	+	+	+	*RMia/rmia*
ACI-191433-04	+	-	-	*rmia/rmia*
ACI-191433-08	+	+	+	*RMia/rmia*
ACI-191433-09	+	-	-	*rmia/rmia*
ACI-191433-X	+	+	+	*RMia/rmia*
ACI-201433-01	+	+	+	*RMia/rmia*
ACI-201433-02	+	+	+	*RMia/rmia*
ACI-201433-03	+	+	+	*RMia/rmia*
ACI-201433-04	+	+	+	*RMia/rmia*
ACI-201433-05	+	+	+	*RMia/rmia*
ACI-201433-06	+	+	+	*RMia/rmia*
ACI-201433-07	+	+	+	*RMia/rmia*
ACI-201433-08	+	+	+	*RMia/rmia*
ACI-201433-09	+	+	+	*RMia/rmia*
ACI-201433-10	+	-	-	*rmia/rmia*
ACI-201433-11	+	+	+	*RMia/rmia*
ACI-201433-12	+	-	-	*rmia/rmia*
ACI-201433-13	+	+	+	*RMia/rmia*
ACI-201433-14	+	-	-	*rmia/rmia*
ACI-201433-15	+	-	-	*rmia/rmia*
ACI-201433-16	+	+	+	*RMia/rmia*
ACI-201433-17	+	-	-	*rmia/rmia*
ACI-201433-18	+	-	-	*rmia/rmia*
ACI-201433-19	+	+	+	*RMia/rmia*
ACI-201433-20	+	-	-	*rmia/rmia*
ACI-201433-21	+	+	+	*RMia/rmia*
ACI-201433-22	+	-	-	*rmia/rmia*
ACI-201433-23	+	+	+	*RMia/rmia*
ACI-201433-24	+	+	+	*RMia/rmia*
ACI-201433-25	+	-	-	*rmia/rmia*
ACI-201433-26	+	+	+	*RMia/rmia*
ACI-201433-27	+	+	+	*RMia/rmia*
ACI-201433-28	+	+	+	*RMia/rmia*
ACI-201433-29	+	+	+	*RMia/rmia*
ACI-201433-30	+	-	-	*rmia/rmia*
Progeny 32 × 02				
Parental 32	-	+	+	*RMia/RMia*
Parental 02	+	-	-	*rmia/rmia*
ACI-183202-01	+	+	+	*RMia/rmia*
ACI-183202-02	-	+	+	*RMia/RMia*
ACI-183202-03	+	+	+	*RMia/rmia*
ACI-183202-04	+	+	+	*RMia/rmia*

Only genotypes available at the time of the analysis were included, as losses of plant material during breeding process prevented the genotyping of complete breeding populations. ND: not determined.

In the progeny derived from the cross between the resistant parental ‘08 – *P. persica*’ and the parental ‘03 – *P. kuramica*’ (**Table 3**), the markers identified resistant homozygous individuals such as ‘ACI-150803-11’ and ‘ACI-150803-20’. The resistant status previously assigned to ‘ACI-150803-20’ based on nematode resistance evaluations was confirmed by all three markers. Conversely, genotype ‘ACI-150803-13’ amplified both resistant- and susceptible-associated fragments, indicating a heterozygous resistant constitution.

To our knowledge, neither resistance to RKN nor the allelic constitution of the *RMia* locus has been previously characterized in this wild species. Therefore, the marker profile obtained for parental 03 cannot be assigned to any currently defined *RMia* genotypic classes (**Table 3**). Despite this limitation, the markers were informative in its progeny and enabled reliable classification of the derived breeding selections. Additional phenotypic and genomic analysis will be required to clarify the genetic constitution of this species at the *RMia* locus.

In the progeny derived from parental ‘14 - P*. dulcis* × (*P. persica* × *P. davidiana*)’ and parental 33 - P*. dulcis* × *P. persica*, the markers identified both heterozygous resistant and homozygous susceptible individuals (**Table 3**). Most selections classified as *RMia*/*rmia* showed amplification of both the susceptible-associated marker RTP-RMia F3/R3 and the resistant-associated markers ACI_9660_2 and NEM_6908_4, whereas susceptible individuals amplified only the susceptible allele. Similar marker profiles were observed in the progeny derived from parental ‘32 - P*. persica*’ and parental ‘02 - P*. bucharica*’, demonstrating the applicability of the marker system across distinct genetic backgrounds.

The ability to distinguish homozygous resistant from heterozygous resistant individuals represents an advantage over previously reported for *RMia*, which primarily allowed identification of resistant genotypes without resolving zygosity (Duval et al., 2025). This information is particularly valuable in breeding programs because homozygous resistant selections can be prioritized as parental to increase the frequency of resistant offspring.

### 3.4 Functional validation of RMia-derived markers against RKN resistance

The association between the developed markers and the RKN resistance was evaluated using a set of advanced breeding selections previously phenotyped against *Meloidogyne incognita*, *M. arenaria* and *M. javanica* (**Table 4**). As the objective of the phenotypic evaluation was to confirm the resistance in selected genotypes before advancing them to the next stage of evaluation, only genotypes previously selected as putatively resistant were included in the assay. Consistently, no homozygous susceptible (*rmia*/*rmia*) genotypes were available for phenotypic validation. Marker analysis classified the evaluated genotypes as either homozygous resistant or heterozygous, and the inferred genotypes were subsequently compared with the corresponding resistant phenotypes.

**Table 4:** Functional validation of the *RMia*-derived markers in advanced *Prunus* breeding genotypes. Resistance responses are indicated as resistant (R) and susceptible (S).

Genotype	*RMia* genotype	Phenotype *M. incognita - M. javanica - M. arenaria*
ACI-150803-11	*RMia/RMia*	R - R/S - R
ACI-150803-20	*RMia/RMia*	R - S - R
ACI-190803-04	*RMia/RMia*	R - R - R
ACI-151406-07	*RMia/rmia*	R - R - R
ACI-163230-03	*RMia/rmia*	R - S - R
ACI-171302-13	*RMia/RMia*	R - S - R
ACI-184742-02	*RMia/rmia*	R - R - R

Within the evaluated breeding panel, marker-inferred genotypes showed complete qualitative agreement with the observed resistance phenotypes against *M. incognita* and *M. arenaria*. All genotypes carrying the resistant allele, either in homozygous or heterozygous conditions, were classified as resistant to both nematode species. These results confirm the strong association between the identified retrotransposon insertion and the *RMia* resistance locus and demonstrate the usefulness of the developed markers for MAS.

In contrast, contrasting responses were observed against *M. javanica*, with some genotypes carrying the resistant allele exhibiting susceptibility to this species. However, this observation is consistent with the known resistant spectrum of *RMia*. Previous genetic studies demonstrated that the resistance to *M. incognita* and *M. arenaria* is controlled by the *RMia* gene located in chromosome 2 of peach (Duval et al., 2013), whereas resistance to *M. javanica* is conferred by the *RMja* gene located in chromosome 7 (Van Ghelder et al., 2010; Duval et al., 2019). Therefore, the response to *M. javanica* is not expected to be predicted by markers associated with *RMia* and should be considered independently.

## 4 Conclusion

The markers developed in this study are based on structural differences between the resistant and susceptible RMia haplotypes, providing a practical tool for the incorporation of *RMia*-mediated resistance into *Prunus* breeding programs. Their codominant nature allows the identification of homozygous and heterozygous resistant individuals, while validation in breeding germplasm and phenotyped selections confirmed their association with the resistance to *M. incognita* and *M. arenaria*. Future work should focus on evaluating marker performance across a broader range of *Prunus* germplasm and combining *RMia*-derived markers with markers linked to *RMja* and *Ma* to facilitate the development of rootstocks carrying multiple sources of nematode resistance.

## Data Availability

The datasets presented in this study can be found in online repositories. The names of the repository/repositories and accession number(s) can be found below: https://www.ebi.ac.uk/ena, PRJEB115460.
